# Whisking Behaviour Reveals Stronger Evidence of Habituation in Homozygous Reeler Mice Compared to Controls

**DOI:** 10.1111/gbb.70049

**Published:** 2026-03-17

**Authors:** Ugne Simanaviciute, Aybeniz Ece Cetin, Julien Guy, Helen Tams, Jochen Staiger, Robyn A. Grant

**Affiliations:** ^1^ Department of Natural Sciences Manchester Metropolitan University Manchester UK; ^2^ Department of Neuroanatomy University Medical Center Göttingen, Georg‐August‐University Göttingen Göttingen Germany

**Keywords:** barrel cortex, behaviour, object exploration, rodent, somatosensory, vibrissae

## Abstract

Reeler mice have a mutation in the reelin gene. As a result, Reeler mice lack cortical layers, yet their brains are still largely functional. However, Reeler mice display strong motor phenotypes, including ataxic gait and tics, and we posit that their whisking behaviour might also be disrupted. We used high‐speed video to film and track whisker movements in 9 adult Reeler mice and 9 age‐matched controls in three whisker movement assessment tasks, including our established novel object exploration and open field tasks, as well as a new open field habituation task. Overall, whisker movements in Reeler mice were highly conserved during the novel object exploration and open field tasks, and they demonstrated all behaviourally relevant whisking features during exploratory contact with an object, including contact‐induced asymmetry, spread reduction, and decreasing whisker speeds following object contact. In the habituation task, whisker angular position and whisker spread decreased between the first consecutive sessions in all mice, suggesting that the animals were less focused on sampling the area as they got more familiar with the environment. However, only Reeler mice were affected by more extensive habituation. We suggest that whisker‐dependent sensory function is surprisingly intact in Reeler mice. However, our observed habituation‐related changes in Reeler mice whisker movements suggest some behavioural differences in these mice, which is a likely result of their disorganised cortex due to reelin deficiency.

## Graphical abstract

In the habituation task, locomotion speed, whisker angular position and whisker spread decreased between the first two consecutive sessions in all mice, suggesting that the animals were less focused on sampling the area as they got more familiar with the environment. However, only Reeler mice were affected by more extensive habituation, up until the fifth session.

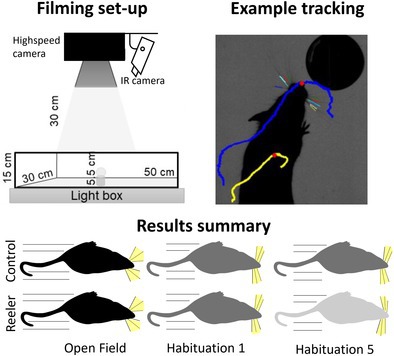

## 1 Introduction

Reelin is a large extracellular matrix protein with a major function in neuronal migration and positioning, especially in the early developing brain [1, 2, 3, 4, 5]. Reeler mice have a mutation in the reelin gene, responsible for the proper production of the protein Reelin [6]. As a result, Reeler mice have a strong disturbance in mainly laminar structures of the brain where neurons inhabit erroneous positions due to disturbed migration. In particular, they have strongly disordered cortical lamination [7], a much under‐developed cerebellum lacking folia, and a disrupted superior colliculus and spinal cord, amongst many other abnormalities [6, 7, 8, 9, 10, 11, 12]. Homozygous Reeler mice are thus used as a model for the developmental disruption of cortical layers. They have also been proposed as a model of lissencephaly type 2 [2] and epilepsy [2, 13].

Up until recently, it has been thought that layers are an important component of how neurons are connected and work in the brain, especially in the cerebral cortex [14, 15]. However, layers in the forebrain are probably not a mandatory component of a normally functioning, high‐performance brain, as can be seen across many non‐mammalian species, such as birds and reptiles. Indeed, Reeler mice still have cortical neurons that are almost entirely functional and topographically connected, despite their lack of distinct cortical layers [16]. This has been especially well‐described in the whisker system of Reeler mice. The topological whisker representations in the Reeler barrel field have been found to be preserved, with individual whisker representations similar to that of wildtype mice, despite the physical barrel structure being strongly deformed [17]. Indeed, whilst the canonical barrel architecture is largely absent, it is replaced by barrel‐equivalent clusters of neurons that are scattered throughout the cortical depth within a barrel‐equivalent‐containing column, thereby preserving the topological whisker map [17, 18]. Wagener et al. [18] investigated experience‐dependent neuronal activity in response to whisker touches during exploratory behaviour in an enriched environment (using c‐Fos). They found that Reeler mice and controls did not show any difference in their activation to whisker touch, suggesting that sensory stimulus‐driven information processing still takes place within the disorganised cortex.

Behaviourally, Reeler mice have been a challenging model to define. They only have slight cognitive impairments in spatial memory and executive functioning [19]. Salinger et al. [20] exposed Reeler mice to a battery of behavioural tests and reported them behaving similarly to control animals during tasks involving olfactory food‐finding, depth perception and acoustic responsiveness. Indeed, basic visual abilities in Reeler mice also remain intact; however, they perform worse in more complex perception tasks, such as orientation discrimination [21]. Therefore, sensory function is likely to remain intact in Reeler mice, despite revealing strong motor phenotypes, including an ataxic gait [22, 23, 24]. They also have deficits in forepaw dexterity, including paw guiding and grasping movements [25]. Interestingly, the facial nucleus, the effector of whisker movement, has also been found to be disrupted in Reeler mice [26]. Therefore, whilst sensory sensitivity and processing may remain intact in Reeler mice, impaired motor control may yet impact the quality of sensory signals they receive and, therefore, compromise behaviour.

A useful way to study this further is by measuring whisker movements. Whiskers make cyclic to and fro movements, called whisking, which occur at 12–25 Hz in mice [27]. As well as whisking, whiskers are precisely controlled to change their symmetry, spread, speed and forces in order to improve the sensory signals they receive. Their movements can be quantitatively tracked, and certain behaviours have been found to be associated with exploration [28]. For instance, exploratory whisking in an open space is associated with large amplitude movements of largely spread‐out whiskers [29]. Object‐related exploratory, or foveal, whisking is associated with a reduction in whisker spread and speed, and an increase in amplitude and asymmetry [30, 31]. A recent study in our lab [28] found whisker movements to be highly conserved in heterozygous Reeler mice compared to control animals, although we did observe significant differences between male heterozygous Reeler and wildtype mice whisker angular positions at 7–9 months. This suggests that precise whisker positioning may be impacted in Reeler mice. However, given the richness in expressed motor patterns of intact whisker movements, a more systematic whisker behaviour study is still needed.

In this study, we set out to investigate whisker movements in Reeler mice in three whisker movement assessment tasks, including our established novel object exploration and open field tasks [28, 32, 33], as well as a new open‐field habituation paradigm with whisker kinematic measurements. Measuring whisker movements complements standard open field habituation tasks by providing a quantitative measure of sensory exploration, over and above simple positioning and path data. The present results give us the first insight that mouse whisker movements and positions change during habituation, with subtle but reliable differences being observed in the Reeler mutant. This may have important implications for behavioural neuroscience, specifically by developing new tasks for the behavioural phenotyping of rodent models and demonstrating how neuronal disorganisation, driven by the Reelin gene mutation, affects behaviour.

## 2 Materials and Methods

### 2.1 Animals

Homozygous Reeler mice were used for all analyses, as they display robust and consistent neuroanatomical and behavioural phenotypes compared to heterozygous animals, which are sometimes even used as controls. To generate cell‐type‐specific fluorescently labelled Reeler mice, two heterozygous Reeler parent lines were established. One parent line consisted of PV‐Cre, VIP‐Cre or SOM‐Cre mice that were homozygous for the respective Cre allele (Cre^+^/Cre^+^) and heterozygous for the reelin mutation (rl^+^/rl^−^). The second parent line consisted of Ai9 tdTomato reporter mice (B6.Cg‐Gt(ROSA)26Sortm9(CAG‐tdTomato)Hze/J, The Jackson Laboratory) that were homozygous for the reporter allele (T/T) and heterozygous for the reelin mutation (rl^+^/rl^−^). Crossing these two lines yielded offspring with tdTomato expression restricted to VIP‐ (vasoactive intestinal peptide), SOM‐ (somatostatin) or PV‐ (parvalbumin) expressing interneurons. From this litter, mice homozygous for either the wild‐type (rl^+^/rl^+^) or the Reeler (rl^−^/rl^−^) allele were selected for experiments, whilst heterozygous animals (rl^+^/rl^−^) were not used. We do not predict the behaviour of the mouse lines (PV‐Cre, VIP‐Cre or SOM‐Cre) to be different, since they only differ for cell type‐specific fluorescent protein expression. However, for consistency, the behaviour of the Reeler mice from different lines were compared and were reassuringly not found to be different. Therefore, results from all Reeler mice lines were combined, and the mice will be referred to from here as Reeler mice only. Data from this investigation can be found in Supplement 1, Figures S1 and S2. Wildtype littermates were used for comparison in the experiments. Mice were group housed in standard cages on a reversed 12 h light/dark cycle (lights on at 7 pm) and with *ad libitum* access to food and water. Due to the demanding breeding scheme of the Reeler mice, it is difficult to produce mice in specific, narrow age groups. Therefore, mice of different litters were pooled and their age ranged from 2 to 8 months—a sample range of representative, healthy, adult mice. However, the effect of age and sex were still included in our statistical analyses (see Statistical analysis section below, and sample number table in Supplement 2, Table S1). The experimental protocol was approved by the Lower Saxony State Office for Consumer Protection and Food Safety (Landesamt für Verbaucherschutz und Lebensmittelsicherheit (LAVES) of Lower Saxony, AZ 33‐19‐42502‐04‐19/3157).

**FIGURE 1:**
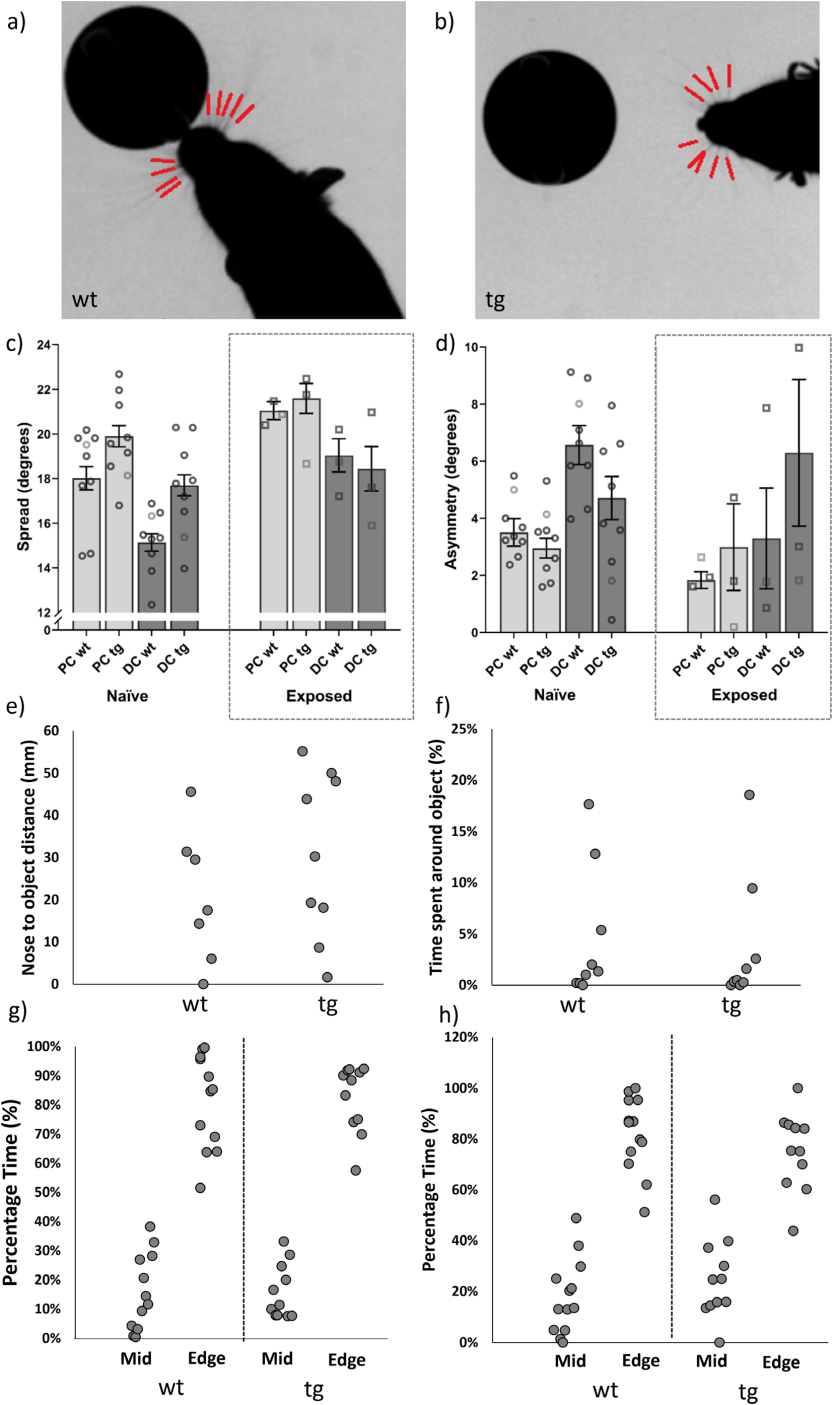
Novel object exploration task results. Top panels show an example video still illustrating the minimum distance to the object for a wildtype (a) and Reeler (b) mouse during the novel object exploration task. ARTv2 LocoWhisk software was used to automatically locate the whiskers (red lines) and detects them on a frame‐by‐frame basis. Both pictures show whiskers at their maximum protraction. Middle panels show whisker movement measurements: Spread (c) and asymmetry (d). Note the increase in asymmetry and a decrease in spread from the PC to DC part of the video clips, representing the robust contact‐related changes in naïve mice. The bars indicate the mean values from all the clips (degrees of freedom calculated from a linear mixed‐effect model), with error bars representing SEM. Data points show mean values for individual mice. PC = pre‐contact, lighter bars, DC = during contact, darker bars, wt = wildtype, tg = transgenic Reeler mice. Note the discontinuous y‐axis in panel (c). The inset panels on the right refer to the small number (*n* = 4) of mice that were exposed to the experimental arena in sessions before data collection. Due to the significant differences in naïve (*n* = 9, round points, left hand panels) and pre‐exposed mice (square points, right hand panels), the pre‐exposed mice were removed from all subsequent analyses. Bottom panels show minimum nose distance measurements to the object (e), the percentage of time around the object (f) and the percentage of time spent in the middle and around the edge of the arena (g) during the novel object exploration task. Panel (h) shows the percentage of time in the middle and round the edge of the arena during the open field task.

The mice were introduced to three different tasks: (i) a novel object exploration, (ii) an open field task (both based on [28]), and (iii) a new open field habituation task. All tasks occurred in the same arena (Perspex rectangular arena, 30 × 50 × 15 cm) with only the object presence and number of sessions changing. The novel object exploration and open field tasks occurred in the same session on the same day. The 22 animals were used throughout the tasks, including 12 Reeler (4 female, 8 male) and 10 wildtype mice (5 female, 5 male). The 18 mice (9 Reeler, 9 WT) undertook the novel object exploration and open field tasks first and six (3 Reeler, 3 WT) undertook the novel open field habituation task first. This meant that six mice had been exposed to the arena for five sessions prior to the novel object exploration and open field tasks (termed previously exposed) and 18 mice were naïve. Results from the previously exposed mice will be presented in the first Results section (Figure 1), but as exposure did significantly affect the behaviour of these animals, they were removed from all analyses, leaving 9 Reeler and 9 wildtype for the full analysis. The order for the experiments of the mice was chosen using randomisation within blocks, balancing for sex, age and strain. Experimenters were not blind to whether the mice were Reeler or wildtype controls during the experimental procedures due to the visible ataxic phenotype of Reeler mice. However, the experimenters were blinded to this during the highspeed video analyses section of the experiment, as the symptoms did not clearly reveal in these short clips.

### 2.2 Experimental Procedures

Experimental procedures were all based on our previously developed whisker filming protocols, detailed in [28, 32]. Briefly, in all three tasks, the arena was illuminated from below using an infrared light box (LEDW‐BL‐400/200‐SLLUB‐Q‐1R‐24 V, PHLOX). The mice were filmed from above with two cameras. Whisker movements were captured using a Phantom Miro ex2 digital highspeed video camera (500 frames per second, shutter speed 1 ms at 640 × 480 pixels). An additional overhead infrared camera (DVcam, 30 fps at 1920 × 1080 pixels) was introduced to record body movements throughout the whole arena for the length of the session. For the novel object exploration task, a Pyrex glass bottle stopper was placed centrally in the arena to facilitate exploratory behaviour for 5–10 min. The bottle stopper was then removed to start the open field task, giving the mice another 5–10 min of open field exploration. Many 1.6 s video clips were collected by a manual trigger when the mouse moved under the highspeed camera, which was positioned centrally over the arena. For the open field habituation task, the same mice were placed in the same open arena (without an object) for 10 min each, making up five sessions over three consecutive days on separate occasions from the previously mentioned tasks. The mice were filmed on the 1st and last (5th) session of the habituation task.

### 2.3 Highspeed Video Analysis

All highspeed video clips were reviewed against the inclusion criteria developed in Grant et al. [33]. For the open field and open field habituation tasks these included that: (i) the mouse was fully in frame, (ii) both whisker sides were visible, and (iii) the head was level, with minimal pitch, roll, and yaw. Only including portions of footage where the mouse has a levelled head is important to ensure accurate two‐dimensional tracking of the whiskers but could have removed episodes of extreme locomotory ataxia. However, whisker movements are unlikely to be interrupted by ataxic gait. For example, when gait is highly affected in SOD1 mice, their whisker movements remained relatively unchanged, since whiskers play a primary role in tactile exploration and are not especially coupled to gait parameters [33]. For the novel object exploration task, clips were sectioned into pre‐contact (PC) and during contact (DC) frames. Therefore, for the novel object exploration task, clip selection criteria additionally included: (iv) the rodent moved towards the bottle stopper in PC, and (v) the whiskers contacted the bottle stopper in DC. Only clips that had > 0.2 s sections of both PC and DC frames were included in the analysis for the novel object exploration task.

The highspeed clips were tracked using the Automated Rodent Tracker (ARTv2) [34], which automatically detects the snout, the centroid of the body and whiskers (Figure 1a,b). More details of ARTv2 and its metrics can be found in [28, 32] and [34]. In summary, the software detects a total of 2–12 whiskers in each frame of video (with around 5–6 on each side being usual, Figure 1a,b). A mean whisker angle is approximated from each frame for all whiskers detected in that frame. Higher whisker angles correspond to more forward‐positioned (protracted) whiskers. Whisker detection was checked by manual inspection of the tracking. The 1–9 clips per mouse were included in data analysis, with a total of 110 open field clips and 73 object exploration clips. In the habituation study, first habituation had 189 video clips and the fifth habituation included 106 clips. All these totals did not include the previously exposed mice. Following clip selection and tracking review, the final animal numbers included for each task can be found in Supplement 2, Table S1, broken down by genotype, sex, line and age.

A number of outcome measures were then obtained from the mean whisker angles calculated by ARTv2, including: mean angular position (the mean whisker angle), asymmetry (the difference in whisker angles between the left and right sides), amplitude (2√2* the standard deviation of whisker angles), mean angular retraction and protraction speeds (the average speed of the backward (negative) and forward (positive) whisker movements, respectively), and whisker spread (standard deviation of all tracked whisker angular positions). For all the measures apart from amplitude, a mean was taken of the right and left whisker measurements to give one value per video clip. Mean locomotion speed was also extracted from the centroid position of the mouse, calibrated to distance using a ruler.

Novel object exploration task clips were assessed to investigate if Reeler mice got as close to the object as wildtype mice. The tape measure tool in Tracker (6.1.5) was used to manually measure the shortest distance from the tip of the nose to the object, once per video clip, when the mouse was the closest to the object. The 152 clips were included in this analysis. As well as quantitatively measuring the distance to the object, the overhead camera was used to extract the percentage of time that the mice spent around the arena walls, in the middle of the arena for both the object exploration and open field tasks, and around the object for the object exploration task. These timings were extracted manually for each mouse, for each session, using BORIS software (v.8.20, [35]), and presented as percentages due to the difference in the total time of each session (Figure 1f,g).

### 2.4 Statistical Analysis

For all whisker and locomotion measurements, each outcome variable (mean angular position, amplitude, asymmetry, mean angular retraction and protraction speeds, spread and locomotion speed) was compared between the control wildtype mice and the mutant/transgenic mice, separately for each task (novel object exploration, open field, open field habituation). For the novel object exploration task, the PC whisker variables were first analysed. Then contact‐related changes in the whisker variables were analysed by subtracting DC from PC (PC‐DC). PC‐DC was chosen, rather than DC‐PC, as it is more intuitive to identify increases in variables during contact as positive, and reductions as negative; in addition, many of the whisking parameters were expected to be higher in PC. To identify main effects in each task, a MANOVA was conducted on the dependent whisker variables, with genotype, age and sex as fixed effects and individual mouse ID as a random effect. In the novel object exploration task, contact was added as a within factor, and in the habituation task, habituation session was added as an additional fixed effect. Effect sizes were approximated using partial eta‐squared (η^2^p), with values of 0.01 classed as a small effect, 0.06 as medium and 0.14 as large. Following this, separate Linear Mixed‐Effects Models (LMEM) (lme4 in RStudio [36]) were conducted on each whisker variable, with genotype, age and sex as fixed effects and individual mouse ID as a random effect. All models calculated F tests on the fixed effects and provided *p*‐values with a significance level of *p* < 0.05. The LMEM was a good fit to our experiment since we could include the video clips as within variables, and our data all conformed to the assumptions of the model, i.e., our residuals were all normally distributed. Each video clip was treated as a within variable since they were not independent. A Kenward‐Rodger's method [37] was used to approximate the degrees of freedom and F‐statistics. Using this method, the degrees of freedom (the experimental units) were automatically calculated to fall anywhere between the number of animals and number of video clips. This is a way to make the most of the small animal sample sizes (9 wildtype and 9 Reeler in most cases) and ensure that the statistical power is robust and generalisable. A sample size of nine animals in each group is also considered usual for studies of this type [28]. The nose distance data were analysed in the same way as individual whisker variables. The overhead video clips were analysed as percentage of time spent in the areas of arena; there was only one overhead video per session, giving only one measure per individual mouse. Therefore, these timings were statistically evaluated using Pearson's *χ* ^2^ tests.

## 3 Results

### 3.1 Novel Object Exploration Task

The novel object exploration task was used to determine if any object‐related motor deficits were present in Reeler mice. Significant differences were found between the previously exposed and naïve animals in PC asymmetry (*t* = 2.168, df = 22, *p* = 0.0413), PC spread (*t* = 2.113, df = 22, *p* = 0.0462), and PC‐DC protraction speed (df = 22, *t* = 3.118, *p* = 0.0050), with spread lower and asymmetry higher in the naïve animals, compared to the exposed (Refer to Figure 1c,d for the associated mean and standard error values; left panel and round points correspond to naïve mice, and right panels with square points to previously exposed mice). However, the data from previously exposed mice could not be analysed separately, as it was such a small sample size. Therefore, they were removed from the sample and only naïve mice were included in all subsequent analyses (including in the subsequent open field and open field habituation tasks). Although the naïve and previously exposed mice are both included in Figure 1 (panels c and d) for viewing.

A MANOVA showed that genotype (*F*(7, 53) = 2.136, *p* = 0.055, *η* ^2^ *p* = 0.220), sex (*F*(7, 53) = 0.727, *p* = 0.650, *η* ^2^ *p* = 0.088) and age (*F*(56,413) = 1.266, *p* = 0.105, *η* ^2^ *p* = 0.147) did not significantly affect the whisker variables in the novel object exploration task. However, contacting the object had a large and significant effect (*F*(7, 53) = 9.229, *p* < 0.001, *η* ^2^ *p* = 0.549). Indeed, the predicted robust contact‐related behaviours were present in both Reeler and wildtype naïve mice, including an increase in amplitude and asymmetry, and a decrease in locomotion speed, retraction speed, protraction speed and spread following object contact. However, there were no genotype, sex or age differences in any (PC‐DC) variables, including (PC‐DC) amplitude, mean angular position, spread, asymmetry, locomotion speed, retraction speed, protraction speed and spread (for all, *p* > 0.05).

Individual linear models found a genotype effect in PC whisker spread [genotype: *F* (1, 12.057) = 5.4403, *p* = 0.03781; sex: *F* (1, 12.057) = 0.2001, *p* = 0.66254; genotype‐sex interaction *F* (1, 12.057) = 2.5175, *p* = 0.13845; age: *F* (3, 9.1696) = 0.5064, *p* = 0.6873] (Refer to Figure 1c for the associated mean and standard error values, sex and age effects are not shown on the figure), with Reeler mice having larger PC spread values than the wildtype mice. However, pairwise comparisons showed no significant differences in post hoc tests (*p* values > 0.05). There were no genotype, sex or age differences in any other PC variables, including PC amplitude, mean angular position, asymmetry, locomotion speed, retraction speed and protraction speed (for all, *p* > 0.05).

When we reviewed the videos, we noticed that in many of the clips the Reeler mice often did not contact the object during the novel object exploration task and stopped just short of contacting the object with their whiskers (Figure 1b), causing many of these video clips to not be included in the analysis. In agreement with this observation, there was a small, significant difference between Reeler and wildtype mice in the nose to object distance measure (*F* (1, 14) = 5.471, *p* = 0.021, *η* ^2^ *p* = 0.038, Figure 1e), with Reeler mice being around 10 mm further from the object than wildtype mice (Reeler: 31 ± 26 mm, Wildtype: 21 ± 26 mm). There were no significant differences in the percentage of time spent around the object, around the walls, or in the middle of the arena between Reeler and wildtype mice in the overhead infrared video clips (Pearson's *χ* ^2^ test: *X* ^2^ = 0.84438, df = 2, *p* = 0.6556, Figure 1f,g).

### 3.2 Open Field Task

**FIGURE 2:**
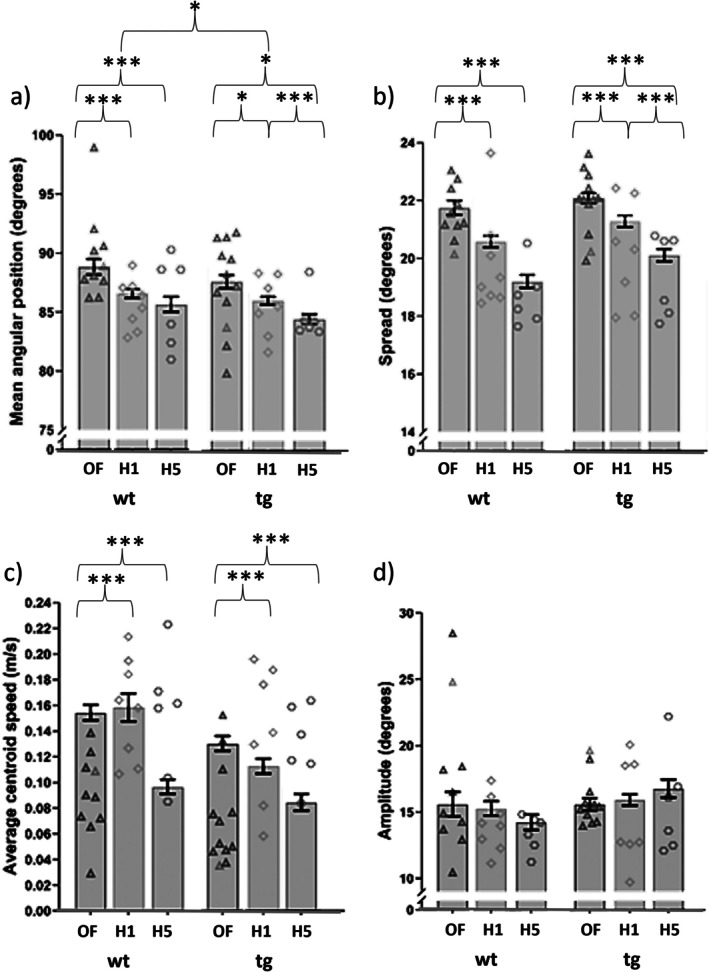
Open field habituation affected whisker and locomotion measures. Significant habituation effects were found in mean angular position (a), spread (b), and locomotion (average centroid) speed (c). Significant habituation effects were also observed in amplitude (d), but there were no significant post hoc test results. The bars indicate the mean values from all the clips (degrees of freedom calculated from a linear mixed‐effect model), with error bars representing SEM. Data points show mean values for individual mice. OF = open field, triangle points, H1 = first habituation, diamond points, H5 = Fifth habituation, hexagon points. Asterisks mark significant values where *p* ≤ 0.05 = *, *p* ≤ 0.001 = ***. wt = wildtype, tg = transgenic Reeler mice. Note the discontinuous *y*‐axis in panels (a), (b), and (d).

The open field task was used to determine if any non‐object related motor deficits were present in the Reeler mice. There were no significant differences in the percentage of time spent around the walls or in the middle of the arena between Reeler and wildtype mice in the overhead infrared video clips (Pearson's *χ* ^2^ test: *X* ^2^ = 1.4516, df = 2, *p* = 0.2282, Figure 1h). There were also no genotype, sex, or age differences in any whisker variables in the open field task, including mean angular position (Figure 2a), spread (Figure 2b), asymmetry, locomotion speed (Figure 2c), amplitude (Figure 2d), retraction speed, and protraction speed (for all, *p* > 0.05).

### 3.3 Open Field Habituation Task

Due to the absence of any whisker movement and position differences in our usual whisker movement tests, we introduced an additional task to our battery. Since we observed differences in naïve and habituated animals to the arena, as well as Reeler mice perhaps distancing themselves from the object, we applied here a novel open field habituation task to assess how well Reeler mice habituate to the experimental arena. Whisker movements were measured in the open field task over a period of five habituation sessions (open field habituation task) and compared to the original open field test. A MANOVA showed that genotype (*F* (7, 354) = 9.716, *p* < 0.001, *η* ^2^ *p* = 0.161) and habituation (*F* (14, 710) = 14.736, *p* < 0.001, *η* ^2^ *p* = 0.225) had large, significant effects on the whisker variables, whereas age (*F* (63, 2520) = 0.253, *p* = 0.986, *η* ^2^ *p* = 0.006) and sex (*F* (7, 354) = 2.482, *p* = 0.116, *η* ^2^ *p* = 0.007) did not significantly affect them. Furthermore, habituation affected the Reeler mice whisker variables more, with larger effect sizes, than the wildtype mice [Reeler: (*F* (14, 390) = 11.488, *p* < 0.001, *η* ^2^ *p* = 0.292); Wildtype: (*F*(14, 308) = 7.080, *p* < 0.001, *η* ^2^ *p* = 0.243)]. Effect sizes and confidence intervals for each whisker variable can be seen in Supplement 3, Table S2 for both Reeler and wildtype mice.

Individual linear models for each whisker variable revealed that genotype (*F* (1, 21.83) = 4.3695, *p* = 0.04845), habituation (*F* (2, 392.30) = 27.0910, *p* < 0.001) and their interaction (*F* (2, 392.30) = 3.7679, *p* = 0.02394) all affected whisker mean angular position. For both Reeler and wildtype mice, the open field mean whisker angular positions were higher than those in the subsequent first habituation session (Reeler: *p* = 0.0251, wildtype: *p* < 0.0001) and those in the fifth habituation session (Reeler: *p* < 0.0001, wildtype: *p* = 0.0009) mice. Reeler mice also had larger mean whisker angular positions in the first habituation session compared to the fifth habituation session (Reeler: *p* = 0.0132). There was no main effect of genotype (*F* (1, 21.99) = 0.0310, *p* = 0.86182), but an effect of habituation (*F* (2, 386.93) = 100.0567, *p* < 0.001) and an interaction with genotype and habituation (*F* (2, 386.93) = 3.4591, *p* = 0.03243) on whisker spread. In both Reeler and wildtype mice, whisker spread was higher in the open field compared to the first and the fifth habituation session (*p* < 0.0001). In Reeler mice, whisker spread was also higher in the first habituation session compared to the fifth (Reeler: *p* = 0.0001). (Refer to Figure 2a for mean angular position and b for spread for the associated mean and standard error values).

Habituation, but not genotype, affected both locomotion speed (*F* (2, 389.35) = 52.7808, *p* < 0.0001), and whisker amplitude [genotype: *F* (1, 21.69) = 0.2716, *p* = 0.60756; habituation: *F* (2, 394.77) = 3.4589, *p* = 0.03242]. For both wildtype and Reeler mice, locomotion speed was higher in the original open field condition, compared to both the first habituation session (wildtype and Reeler: both *p* < 0.0001) and fifth habituation session (Reeler: *p* = 0.0001, wildtype: *p* < 0.0001) (Refer to Figure 2c,d for the associated mean and standard error values). Whisker amplitude revealed no significant post hoc results. There were no genotype nor habituation differences in asymmetry, retraction speed or protraction speed (for all, *p* > 0.05). There were no significant sex or age differences in any variables, including locomotion speed, amplitude, mean angular position, spread, retraction speed and protraction speed (for all, *p* > 0.05).

## 4 Discussion

As a prerequisite of active sensing, rodent whiskers and their movements have been previously subjected to rigorous study [31, 38, 39, 40]. Here, we confirm part of these findings and extend them to a mutant mouse suffering from central motor control deficits at several levels of the neuraxis [24, 25] to test how much whisking and other locomotor behaviours might be affected. Overall, we observed that whisker movements in Reeler mice were highly conserved during the novel object exploration and open field tasks, not differing from the wildtype mice. Indeed, the Reeler mice revealed the precise contact‐related whisker control behaviours that we would expect during exploratory object contact, including increasing amplitude and asymmetry, and decreasing retraction speed, protraction speed and spread following contact. This suggests that the neuroanatomical and motor abnormalities caused by reelin deficiency in Reeler mice do not affect their general whisker movements and the acquisition of sensory information. However, we did observe changes in whisker behaviour during the open field habituation task, in both the wildtype and Reeler mice, with habituation strongly affecting the Reeler mice in every tested session. This manifested as reductions in whisker angular position, spread and locomotion speed, suggesting a reduction in the exploratory behaviours we would usually associate with open field exploration, during habituation. This is the first description of whisker movement changes during a habituation task and may relate to the more subtle deficiencies in spatial memory and executive functioning, first noticed in Reeler mice by Goldowitz and Koch [19].

### 4.1 Habituation

Effects of habituation to the arena on whisker movements were first observed during the object exploration task. The expected exploratory behaviour of a mouse upon contacting a new object is to increase whisker asymmetry and reduce whisker spread and whisker speed [31, 41, 42]. However, all the previously exposed mice (including both wildtype and Reeler mice) had lower PC asymmetry and higher PC spread than naïve mice. They also had lower (negative) PC‐DC protraction speeds than naïve mice, meaning that protraction speed was not reduced following a contact. This might suggest a lack of interest or attention in the arena and object following ‘successful’ habituation to the space. Indeed, these behaviours have all previously been suggested to be related to attention [29, 43].

Following this, we observed significant differences in the open field habituation task. In both Reeler and wildtype mice, mean angular position, spread, and locomotion speed all generally decreased over the habituation period, from the first open field session to the first and fifth habituation sessions (Figure 2). This meant that, following habituation, all animals were moving slower and were holding their whiskers further back and less spread out. This means that these animals may miss contacting an object within the environment (since their whiskers are bunched together and occupying less space) and that any contacts that do occur will be closer to the face [29], perhaps even leading to collisions. Indeed, Arkley et al. [29] suggested that increasing whisker angular position by around 10° in rats may increase the distance to collision by around 6 mm. We observed only a reduction of a couple of degrees in mean angular position, but this could still have a significant impact in collision distance for smaller, fast‐moving mice. Moreover, the Reeler mice showed even stronger habituation (with larger effect size values), and significant behavioural changes also occurred between the first and fifth habituation sessions. Both whisker angular position and whisker spread decreased consistently between the open field and the first and fifth habituation sessions, suggesting that the animals were even less focused on the area ahead of themselves as they got more familiar with the environment [29], further suggesting a decrease in attention during exploration following habituation to the arena [29, 43]. Although these differences are only subtle, active sensing by mouse whiskers only really involves two sensory signals to function: (i) a reafferent signal of motor activity that encodes angle and phase of the whiskers in the whisk cycle; and (ii) an ex‐afferent signal that encodes a contact [44]. We posit that both these might be affected here during habituation, and even more so in the strongly habituated Reeler mice. For instance, reducing whisker spread during open field whisking may reduce the likelihood of object contact. Reducing whisker angular position will affect the reafferent signal, and maybe even the phase of the whisk cycle and the phase of the contact. Furthermore, sensory processing changes as a mouse switches from quiet wakefulness to exploratory whisker behaviour [45] and we do not yet understand what a reduction in exploratory behaviour means for sensory processing. Nevertheless, these observed changes during habituation will probably affect both the likelihood of environmental contact and the resulting sensory processing of any contact.

Habituation over subsequent sessions has never been studied using whisker movements before, and we have not previously observed any habituation changes during the same session, despite often looking for it (Unpublished observation). Arkley et al. [29] trained sighted rats using food rewards to travel down a corridor with changing object positions over subsequent sessions and measured their whisker positions. They also found that increasing familiarity to the experimental set up resulted in a reduction in whisker spread. However, they also noted increased locomotion speeds, more forward positioned whiskers, and decreased whisker amplitudes in the latter sessions, which is the opposite of our findings and likely points to differences between the two tasks. Indeed, in Arkley et al.'s [29] task, the rat switched from locomoting to focussed running for the food reward, with the whiskers protracted forward to act as collision detectors during their fast locomotion episodes [29]; by contrast, our mice generally explored less, by locomoting slower, and holding their whiskers further back, since there was no reward associated with the task. Nevertheless, running multiple sessions and observing whisker movement changes during a habituation period is a useful supplementary task to add to the usual object exploration and open field whisker protocols. Furthermore, care needs to be taken if animals are undertaking multiple behavioural assessments to always ensure the same order of tasks for each individual to make sure that habituation does not have an effect on other tasks.

### 4.2 Novel Object Exploration and Open Field Tasks

Due to the visible ataxia and motor impairments that Reeler mice exhibit [2, 6, 46], it was surprising to see no differences between Reeler and wildtype mice, neither in locomotion speed nor in any whisker movements in the open field task. However, one selection criterion for tracking open field clips was that mice must have their heads fairly level with the floor. This presents the possibility that ataxic episodes were not included in the analyses. Previous investigations have not observed whisker movements to be altered alongside extremely altered gait, since quadrupedal locomotion and whisker movements are not especially well‐coupled [33]. However, future studies could measure whisker movements during episodes of head pitch, roll, and yaw, by filming in three dimensions to account for the changes in head movement, which is a much more challenging set‐up, and makes imaging particularly difficult.

Using our standard novel object exploration task, only one whisker measure—pre‐contact spread—was found to be affected in Genotype and Sex comparisons; however, this was only a small effect, with no significant differences revealed in post hoc tests. Our additional observations suggested that Reeler mice often did not contact the novel object. However, Reeler mice did not significantly differ from wildtype mice in the time spent in the middle of the arena, around the walls or around the object, although the nose to object measurement was slightly larger in Reeler mice, compared to the wildtype mice, by around 1 cm. On the whole, we tend to agree with Guy and Staiger [16], that the whisker sensory function and associated brain control areas are probably mostly intact in Reeler mice. We also observed no significant sex, age or line effects. Whilst in other mouse models, such as those for Alzheimer's disease, Amyotrophic Lateral Sclerosis and Huntington's Disease, we would expect the disease model to progress with age [32, 33, 47], Reeler mice represent a developmental disorder; therefore, we would not really expect differences in adult mice of different ages. We know that our usual open field and novel object exploration tasks are sensitive enough to detect differences in background strain [28], age [32, 47] and sex [28, 48]; hence, we believe that whisker behaviour must be very similar between the strains, ages and sexes in these mice. Although we have previously spotted these differences in mice with a similar sample size to this study [28, 32, 47], having a larger sample size might give us further insights.

However, we did observe changes during habituation, which may indicate cognitive, attentional, or memory differences in the Reeler mice and is likely to affect whisker sensing in these animals. We would expect these findings to be transferrable across different rodent species, although since some whisker behaviours, such as spread reduction, are only found in rodents [42], we might not expect the exact same results across all mammals with moving whiskers. Nevertheless, we suggest that general cognitive and attentional deficits are likely to be a common symptom of a disorganised cortex caused by reelin deficiency in mammals, including humans [49].

### 4.3 Wider Implications

Overall, we present here a new task for studying habituation in an open field arena that has implications across behavioural neuroscience. In our study, we show that all mice showed less interest or attention in the arena following habituation. This presents in a reduction in exploratory behaviours, including the mice moving slower whilst holding their whiskers further back and more spread out. The Reeler mice showed even stronger habituation, with significant behavioural changes occurring between all the measured habituation sessions. Indeed, tracking whisker movements during such a task provides quantitative measurements that are precise enough to be able to detect a behavioural phenotype in Reeler mice, which is a subtle and challenging behavioural model. The small sample size we have access to here may question the statistical power of some of our findings; revisiting the study with a larger and more focused cohort might reveal further insights into whisker deficits in Reeler mice. In terms of generalising this work, our task can be applied to any rodent model but is probably the most useful for characterising rodent models with more subtle behavioural phenotypes, such as subchronic phencyclidine models of schizophrenia [50], maternal immune activation offspring [48], Parkinson's disease [28] and stroke [28]. We believe that measuring whisker movements is a promising quantifiable approach to characterising behaviour and will be useful for describing rodent behaviour in more detail in future studies.

## Funding

This work was supported by the German Academic Exchange Service (57552336).

## Ethics Statement

The study was conducted in accordance with the Declaration of Helsinki and approved by the Lower Saxony State Office for Consumer Protection and Food Safety (Landesamt für Verbaucherschutz und Lebensmittelsicherheit (LAVES) of Lower Saxony, AZ 33‐19‐42502‐04‐19/3157).

## Conflicts of Interest

The authors declare no conflicts of interest.

## Supporting information


**Supplementary 1.** Comparison of mouse lines.
**Supplementary 2**. Animal numbers and breakdown of genotype, sex, line and age for each task.
**Supplementary 3**. Parameter Estimates of habituation task.

## Data Availability

Data is available at: www.github.com/usimana/Reeler_mice. Video clips are available upon request.
