# Supplementary material for: Whisking Behaviour Reveals Stronger Evidence of Habituation in Homozygous Reeler Mice Compared to Controls
Source: Genes Brain Behav. 2026 Mar 17;25(2):e70049. doi: 10.1111/gbb.70049 (PMC13140730; doi:10.1111/gbb.70049)
Supplement: Supplementary file 1 — Supplementary 1. Comparison of mouse lines. Supplementary 2. Animal numbers and breakdown of genotype, sex, line and age for each task. Supplementary 3. Parameter Estimates of habituation task. [file GBB-25-e70049-s001.pdf]

Supplement 1: Comparison of mouse lines.

A Linear Mixed-Effects Model (LMEM) (lme4 in RStudio) was adopted to analyse the fixed effects of genotype and line and the random effect of individual mouse ID on all PC and PC-DC whisker variables. There was no effect of line (VIP, PV or SOM) on any PC or PC-DC metric (all ps>0.05).

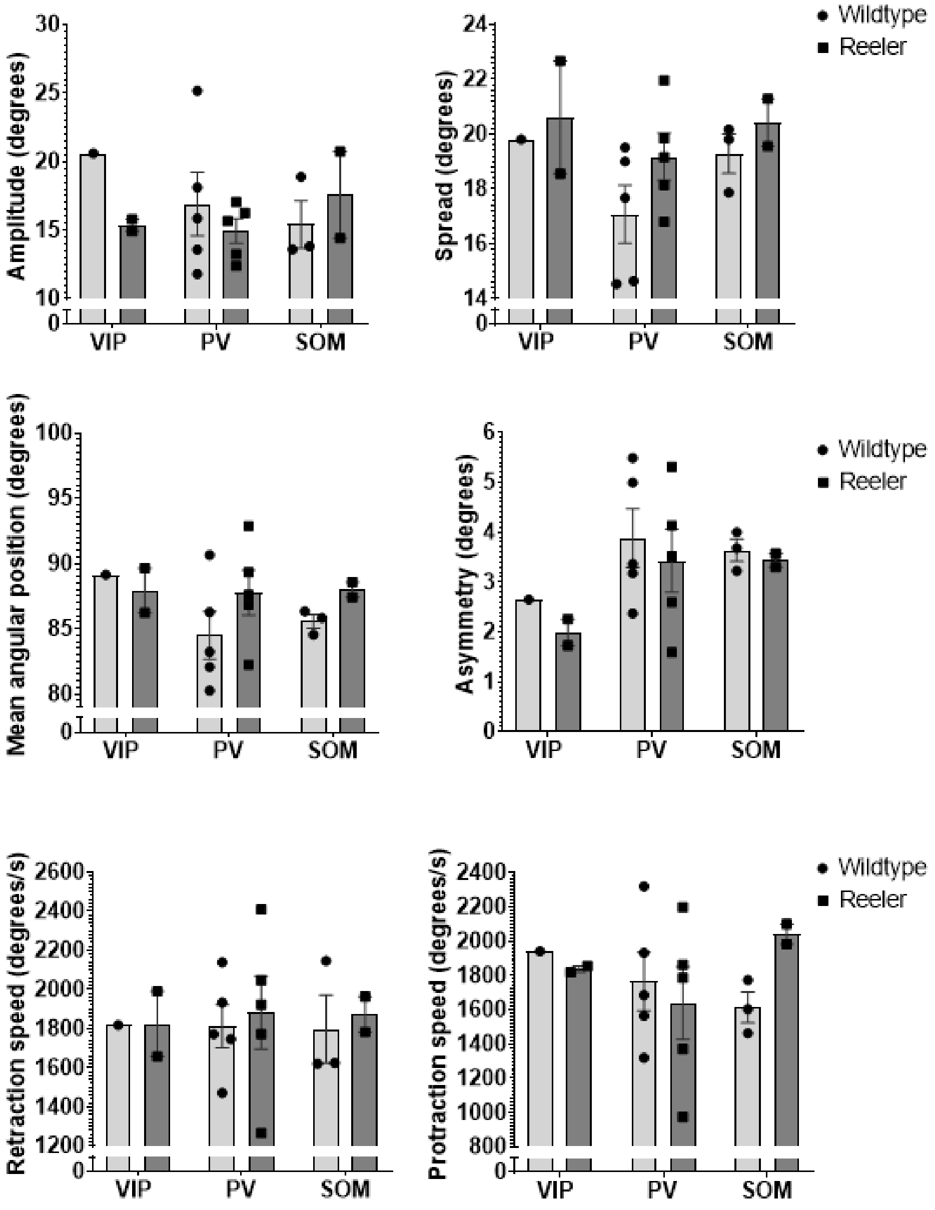

Figure S1: PC data comparing mouse lines. There was no significant difference between the lines VIP, PV or SOM in the PC (pre-contact) metrics.

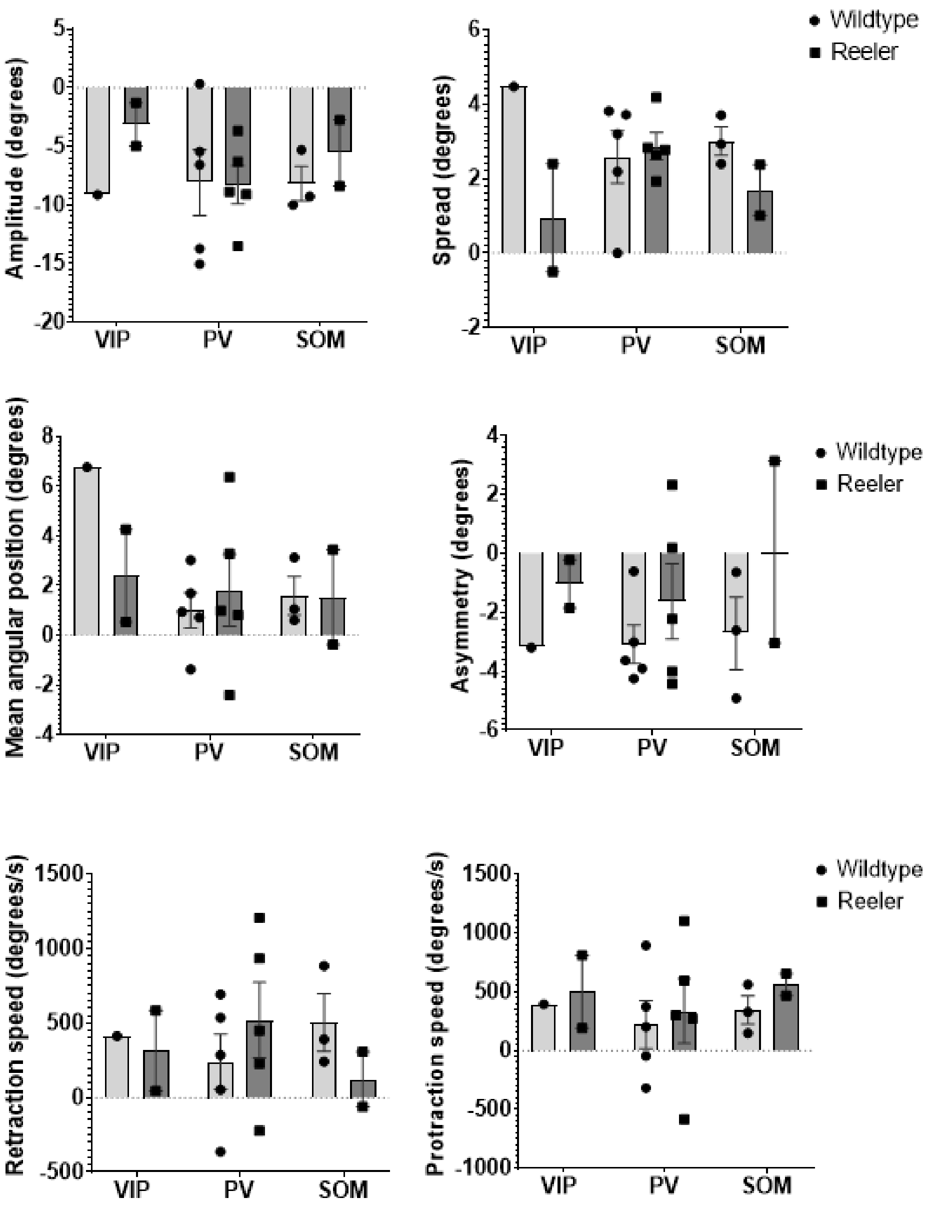

Figure S2. PC-DC data comparing mouse lines. There was no significant difference between the lines VIP, PV or SOM in the PC-DC (pre-contact minus during contact) metrics.

Supplement 2: Animal numbers and breakdown of genotype, sex, line and age for each task.

After clip selection, the breakdown of animal numbers for genotype, sex, line and age for each task can be seen below. The previously exposed animals were not included in any analyses.

Table S1 Animal numbers for each task, including the breakdown for genotype, sex, line, age and previous exposure to the arena.

Open field				
Genotype	12 reeler	10 wildtype		
Sex	13 male	9 female		
Line	6 VIP	6 SOM	10 VP	
Age	8 mice<br>2 months	4 mice<br>3 months	6 mice<br>4.5 months	4 mice<br>8 months
Previous exposure	18 naïve	4 previously exposed		
Novel object exploration				
Genotype	9 reeler	9 wildtype		
Sex	12 male	6 female		
Line	3 VIP	5 SOM	10 PV	
Age	8 mice<br>2 months	4 mice<br>3 months	5 mice<br>4.5 months	1 mouse<br>8 months
Previous exposure	18 naïve	0 previously exposed		
1st Habituation				
Genotype	10 reeler	11 wildtype		
5th Habituation				
Genotype	9 reeler	8 wildtype		

Supplement 3: Parameter Estimates of habituation task

Table S2, Confidence intervals (CI) and effect sizes (partial eta-squared η^2p) for the genotype and habituation effects on the measured whisker variables.

		Reeler			Wildtype		
Dependent Variable	Parameter	Lower CI	Upper CI	η^2p	Lower CI	Upper CI	η^2p
Locomotion Speed	Intercept	71.310	97.740	.417	80.466	112.861	.439
	[Hab=1st]	28.956	62.596	.115	37.524	77.717	.153
	[Hab=5th]	9.638	46.867	.039	38.183	85.310	.131
	[Hab= OF]	N/A					
Amplitude	Intercept	14.458	16.683	.774	14.084	17.087	.703
	[Hab=1st]	-1.071	1.761	.001	-2.183	1.543	.001
	[Hab=5th]	-.375	2.758	.010	-3.533	.836	.008
	[Hab= OF]	N/A					
Whisker Angles	Intercept	86.764	88.630	.994	87.688	89.987	.992
	[Hab=1st]	-2.807	-.433	.032	-3.697	-.844	.053
	[Hab=5th]	-4.491	-1.863	.093	-4.855	-1.510	.074
	[Hab= OF]	N/A	.	.	.	.	.
Asymmetry	Intercept	2.424	3.550	.330	1.949	3.114	.293
	[Hab=1st]	-.768	.666	.000	-.302	1.144	.007
	[Hab=5th]	-.552	1.035	.002	-.400	1.295	.006
	[Hab= OF]	N/A					
Retraction Speed	Intercept	1437.763	1733.053	.669	1320.442	1640.747	.653
	[Hab=1st]	-33.356	342.486	.012	-11.071	386.331	.019
	[Hab=5th]	10.059	426.001	.019	-74.578	391.385	.010
	[Hab= OF]	N/A					
Protraction Speed	Intercept	1486.628	1777.589	.688	1408.081	1715.408	.694
	[Hab=1st]	-149.637	220.695	.001	-127.985	253.313	.002
	[Hab=5th]	-124.013	285.831	.003	-114.007	333.074	.005
	[Hab= OF]	N/A					
Spread	Intercept	21.643	22.523	.978	21.257	22.248	.977
	[Hab=1st]	-1.360	-.241	.035	-1.785	-.555	.074
	[Hab=5th]	-2.594	-1.355	.151	-3.272	-1.830	.216
	[Hab= OF]	N/A					
